# Face-to-Face and Tele-Consults: A Study of the Effects on Diagnostic Activity and Patient Demand in Primary Healthcare

**DOI:** 10.3390/ijerph192114119

**Published:** 2022-10-29

**Authors:** Lourdes E. Barón-Miras, Antoni Sisó-Almirall, Belchin Kostov, Encarna Sánchez, Silvia Roura, Jaume Benavent-Àreu, Luis González-de Paz

**Affiliations:** 1 Department of Preventive Medicine and Epidemiology, Hospital Clínic de Barcelona, 08036 Barcelona, Spain; 2 Consorci d’Atenció Primària de Salut Barcelona Esquerra (CAPSBE), 08028 Barcelona, Spain; 3 Primary Healthcare Transversal Research Group, Institut d’Investigacions Biomèdiques August Pi i Sunyer (IDIBAPS), 08036 Barcelona, Spain; 4 Department of Medicine, Faculty of Medicine and Health Sciences, Universitat de Barcelona, 08036 Barcelona, Spain; 5 Department of Statistics and Operations Research, Universitat Politècnica de Catalunya (UPC), 08034 Barcelona, Spain

**Keywords:** COVID-19, physician–patient relations, primary healthcare, telemedicine, pandemics, remote consultation

## Abstract

Primary healthcare services have changed from face-to-face to tele-consults during the two COVID-19 years. We examined trends before and during the COVID-19 pandemic years based on groups of professionals, patient ages, and the associations with the diagnostic registry. We analyzed proportions for both periods, and ratios of the type of consults in 2017–2019 and 2020–2021 were calculated. The COVID-19 period was examined using monthly linear time trends. The results showed that consults in 2020–2021 increased by 24%. General practitioners saw significant falls in face-to-face consults compared with 2017–2019 (ratio 0.44; 95% CI: 0.44 to 0.45), but the increase was not proportional across age groups; patients aged 15–44 years had 45.8% more tele-consults, and those aged >74 years had 18.2% more. Trends in linear regression models of face-to-face consults with general practitioners and monthly diagnostic activity were positive, while the tele-consult trend was inverse to the trend of the diagnostic registry and face-to-face consults. Tele-consults did not resolve the increased demand for primary healthcare services caused by COVID-19. General practitioners, nurses and primary healthcare professionals require better-adapted tele-consult tools for an effective diagnostic registry to maintain equity of access and answer older patients’ needs and priorities in primary healthcare.

## 1. Introduction

In 2020 and 2021, high- and middle-income countries promoted tele-consults in primary healthcare (PHC) services to minimize COVID-19 transmission, maintain lockdown directives and social distancing measures, and ensure healthcare [1]. Consults by telephone or on the internet have doubled or there was a 50-fold increase in some countries with well-established PHC [2].

Before the COVID-19 outbreak, national healthcare services planned tele-consults to enhance patient access to PHC professionals through digital technologies (websites and specific software), allowing remote contact with general practitioners (GP) and nurses [3,4]. Stakeholders and policymakers saw tele-consults as an alternative to face-to-face consultations to resolve the rising demand for PHC services. However, although telephone consults were frequent and telemedicine tools were available, GPs were pessimistic about their daily use [5]. A UK pilot study before the COVID-19 pandemic showed that tele-consults were efficient in managing small-scale clinical issues, such as administrative requests, repeat prescriptions, and test results [6,7]. However, whether in its basic form of telephone consults, or the more technological guise of video-conference meetings, there were significant challenges and weaknesses to overcome in PHC; tele-consults increased the overall GP workload, many patients required a subsequent face-to-face consult for the physical examination, and the total time used per patient was higher than with face-to-face consults [8,9]. 

To cope with the COVID-19 outbreak, PHC management imposed significant changes in patient care pathways, and GP and nurse planning, enabling stations only for COVID-19 consults, without incorporating more PHC professionals [10]. Tele-consults replaced habitual face-to-face consults; the reduction in physical consults was the most pronounced in respiratory, ophthalmological, digestive, and ear-related diagnoses. The registry of diagnoses of cardiovascular diseases, diabetes, cancer, and the most prevalent chronic diseases decreased by up to 50% in the first pandemic year [11,12]. At the beginning of the first COVID-19 wave (March to May 2020), a decrease in GP diagnostic registry activity may have been linked to lockdowns, fear of COVID-19, and re-organization of PHC services; however, after two years, tele-consult schemes seem to be widely accepted by healthcare authorities [13]. 

The potential benefits of tele-consults might pose barriers to equity; PHC contact with tele-consults might be difficult for older patients, and those with poor technology skills or no access might encounter problems [13]. Furthermore, not all PHC professionals (i.e., GPs, PHC nurses, and social workers) are trained to use tele-consults [14]. Some PHC professionals require face-to-face consults to detect health problems [6]. However, evaluations before and during the pandemic were not made. 

The consequences of the transition from PHC using only face-to-face consults to a mixed system with tele-consults, compared with previous years, are unclear [15]. In Catalonia, a decrease in clinical diagnoses and a drop in patient follow-up has been reported [12,16]. Pandemic PHC tele-consults schemes might have a large impact on health, with models estimating thousands of years of life lost for underdiagnosed conditions, such as cancer [17,18,19]. Therefore, this study compared face-to-face and tele-consults in PHC during the COVID-19 pre-pandemic (2017–2019) and pandemic (2020–2021) periods and studied the association with the frequency in the diagnostic registry of the most prevalent diseases in the PHC setting.

## 2. Materials and Methods

### 2.1. Research Question, Study Design and Aims

This retrospective, observational study was based on visit ratios for the total number of visits, the number of visits with healthcare professionals, and the number of visits by age groups. The monthly frequency of face-to-face and tele-consults in PHC during the pandemic period was compared with the registered number of new diagnoses of the most common disease diagnoses treated in PHC and the number of new and confirmed COVID-19 cases in the study area. 

### 2.2. Study Setting

The study population included patients from three PHC centers in Barcelona city, which provide PHC services within the framework of Spain’s universal and public healthcare coverage. PHC is the first level of contact of patients with the Spanish National Health System. In Spain, PHC is accessible in centers in the community and cares for the most prevalent diseases, conditions, and health problems. When patients require further follow-ups in PHC or other clinical settings, PHC physicians coordinate referrals (i.e., hospital- or disease-oriented healthcare services). PHC centers are the primary source for healthcare services; 72% of the population living in the area assigned to the three PHC centers used PHC services during the study period. The population of the area has a medium–high socioeconomic status and is one of the oldest areas of Barcelona in terms of age, with a mean of 13% of the population being aged ≥75 years, reaching 27% in some districts [20]. The assigned population aged >15 years assigned to the PHC centers during the pre-pandemic period was 86,871, rising to 87,478 during the pandemic.

### 2.3. Data Sources and Variables

Data sources included the Primary Care Services System of Information of Catalonia (SISAP), a centralized database that contains electronic health records of PHC activity, and the COVID-19 open-access data from the Health Ministry of Catalonia [21]. 

We collected data on the total number of consults by all types of PHC professionals, including GPs, PHC adult nurses, PHC pediatricians and pediatric nurses (in patients aged <15 years), social workers, and dentists. Social workers in PHC centers assess the social affairs related to health (e.g., recognition of disability and dependence and gender-based violence management), while dentists provide urgent dental services and deliver health education to prevent dental diseases. The type of visit was defined as face-to-face, which comprised in-person visits, in-center and at-home visits, or tele-consults, which were phone calls, video chats or physician consults of electronic health records to evaluate diagnostic tests, patients’ medication updates or other non-face-to-face consults with administrative tasks. 

We defined four age groups, which were as follows: 15–44 years, 45–64 years, 64–74 years, and ≥75 years. Patients aged ≤14 years were excluded from the age group comparisons due to a lack of complete data. 

The PHC electronic health record in Catalonia uses the International Classification of Diseases 10 (ICD-10) to code the diagnostic registry. To focus on PHC activity, we compiled a list of the most frequent PHC diagnoses, including cardiovascular disease, diabetes, cancer, psychiatric disorders, and chronic diseases. COVID-19 diagnoses were excluded. Online Supplementary Table S1 shows a list of the diseases analyzed and their ICD-10 codes. COVID-19 cases were defined by a positive PCR or rapid antigen test (RAT) and epidemiologically-confirmed cases; ELISA and rapid immunity tests were not included [21].

### 2.4. Statistical Analysis

The type of consult was categorized as face-to-face or tele-consult and the proportions for each period were computed using the type of consult as the numerator and total visits as the denominator. Percentages were calculated according to the PHC professional group. The percentage change in the type of consult between the two periods was calculated using the difference between the means of 2020–2021 and 2017–2019 in telemedicine. Ratios were computed using the mean of consults for 2020–2021 as the numerator and the mean consults from 2017–2019 as the denominator. Ratios were computed according to PHC professional groups and age groups. 

Trends in the diagnoses made by GPs during the COVID-19 period were examined using counts of new monthly diagnoses (from March 2020 to December 2021), and the number of consults with a GP (face-to-face and tele-consults). We excluded other healthcare professionals (e.g., PHC nurses) because they do not often register new diagnoses in the electronic health record. All counts (diagnostic, GP consults, and COVID-19 cases) were rescaled to 0–100, using the monthly value multiplied by 100 and divided by the maximum value from January 2020 to December 2021. For COVID-19 cases, we excluded the first 2 months of 2020, as there were no reports.

We graphically analyzed the trends in face-to-face, tele-consult, and GP diagnostic activity in the framework of the number of new COVID-19 counts of patients with a positive diagnostic test for COVID-19 from March 2020 onwards. Linear regression models from March 2020 to December 2021 (pandemic period) were used to examine time trends, with months as the explanatory variable and face-to-face, tele-consults and new diagnoses as outcomes. Trends were considered significant when the slope of the trend was not equal to zero and had a significant *p*-value (<0.05). Estimates of the slopes of the models gave a numerical interpretation of the tendency of the line, specifically the mean decrease (negative slope) or increase (positive slope) of the type of consults by month. We calculated the R^2^ associated with the regression models. All analyzes were carried out using R v.4.1.2 [22].

### 2.5. Ethical Approval

The study adheres to the Declaration of Helsinki statement, the European General Data Protection Regulations and Spanish laws on Data Protection and Guarantee of Digital Rights. All data used were aggregated and anonymized. Therefore, while no ethical approval was required, the data protection commissioner of the PHC services authorized their scientific use.

## 3. Results

### 3.1. Type of PHC Consults before and during the COVID-19 Years

**Figure 1:**
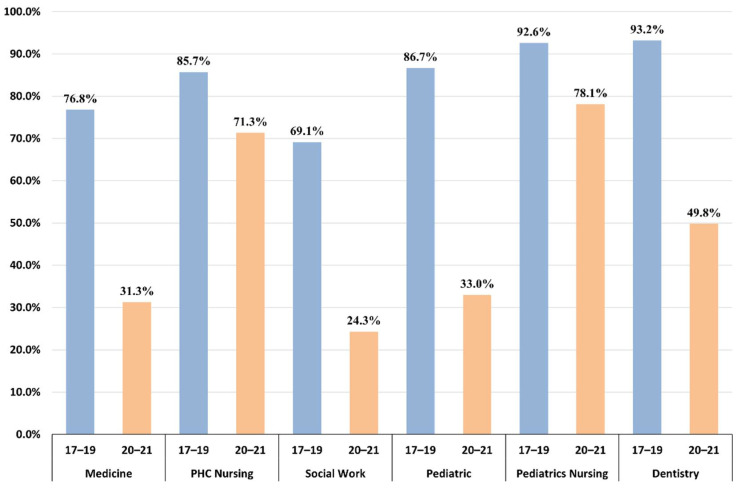
Proportions of face-to-face consults in 2017–2019 and 2020–2021 by type of PHC professional.

Total consults in 2020–2021 increased by 24% compared with the mean in 2017–2019 (from 511,849 to 636,701). Before the COVID-19 pandemic, 80% of PHC consults were face-to-face, but this fell by 34.22% in 2020–2021. Changes in the type of consults differed across PHC professionals; Table 1 shows the mean of yearly consults in 2017–2019 in the three PHCs and ratios with 95% confidence intervals. Total mean consults increased for all PHC professionals during 2020–2021, except for PHC social workers (ratio: 0.92; 95% CI: 0.89 to 0.95). Face-to-face consults with GPs (ratio: 0.44; 95% CI: 0.44 to 0.45) and pediatricians (ratio: 0.81; 95% CI: 0.8 to 0.83) fell, while tele-consults were ten times higher (ratio: 10.78; 95% CI: 10.4 to 11.18). PHC nurses and pediatric nurses maintained face-to-face consults (ratio: 1.12; 95% CI: 1.11 to 1.13 and 2.37; 95% CI: 2.33 to 2.42, respectively). However, tele-consults with PHC adult nurses increased, rising two-fold (from 20,321 to 54,933) and tele-consults with PHC pediatric nurses rose seven-fold (ratio: 7.03; 95% CI: 6.59 to 7.51). The bar graph in Figure 1 shows the proportions of face-to-face consults in the two periods and by type of PHC professional.

### 3.2. Changes in the Visit Patterns by Age Groups

The distribution of consults by age groups were as follows: 25% (15–44 years), 25% (45–64 years), 17% (65–74 years), and 33% (≥75 years). Ratios of consults across age groups before and during the pandemic period showed that consults increased in the 65–74 and ≥75 years age groups by 13% and 12%, respectively, compared with 74% in the 15–44 years age group. Younger age groups had more consults; the ratio of tele-consults was 9.45 for patients aged 15–44 years, compared with 2.3 in patients aged >74 years. These differences were shown across PHC professionals, where increases in GP consults in the 65–74 and >74 age groups were small, with ratios of 2.88 and 2.26, respectively, compared with younger groups (ratios of 8.6 and 5.88, respectively). Greater differences across age groups were observed for PHC adult nurse consults; a ratio of 1.06 in face-to-face visits for the >65 years age group, compared with 1.93 in the 15–44 years age group (Table 2), was reported. 

### 3.3. Trends of Type of Consults, GP Diagnostic Activity, and COVID-19 during the Pandemic Period

**Figure 2:**
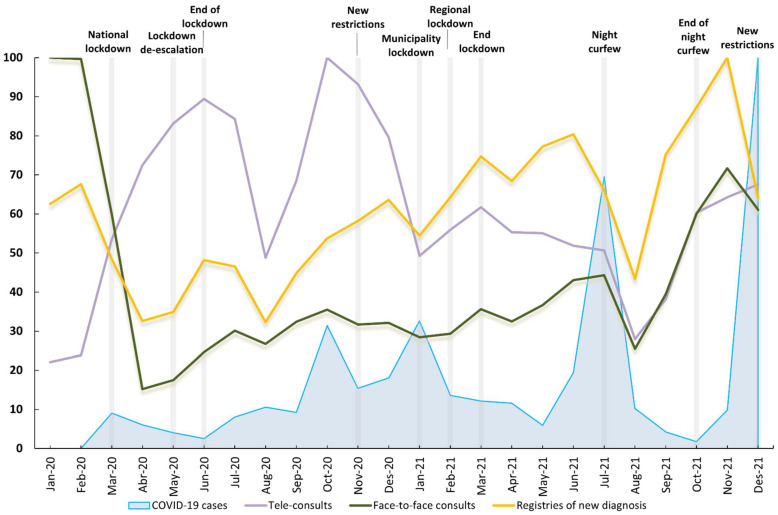
New diagnoses (excluding COVID-19 cases), tele-consults, face-to-face consults and number of COVID-19 confirmed cases in the study area from January 2020 to December 2021.

Figure 2 shows PHC diagnostic registry activity (excluding COVID-19 cases) carried out by GPs, including tele-consults and face-to-face consults, and the number of COVID-19 cases in the three PHC centers; all trends are standardized to a 0–100 scale. The graph shows the significant stance taken by public health authorities due to COVID-19 from March 2020 to December 2021. Face-to-face consults had the same rises and falls as the trend in the registry of new disease diagnoses. In November 2021, the number of new diagnoses peaked after a decrease in March 2020. Tele-consults showed the same falls in August in the two years (due to holidays). However, the rise coincides with the public restrictions imposed (i.e., lockdowns) and the six COVID-19 epidemic waves (beginning in March 2020, June 2020, September 20, December 20, May 21, and October 21). Tele-consults peaked in October 2020 and fell by almost 60% over subsequent periods, with no increase during the periods with higher diagnostic activity. Supplementary Table S2 shows the original and rescaled values. 

**Figure 3:**
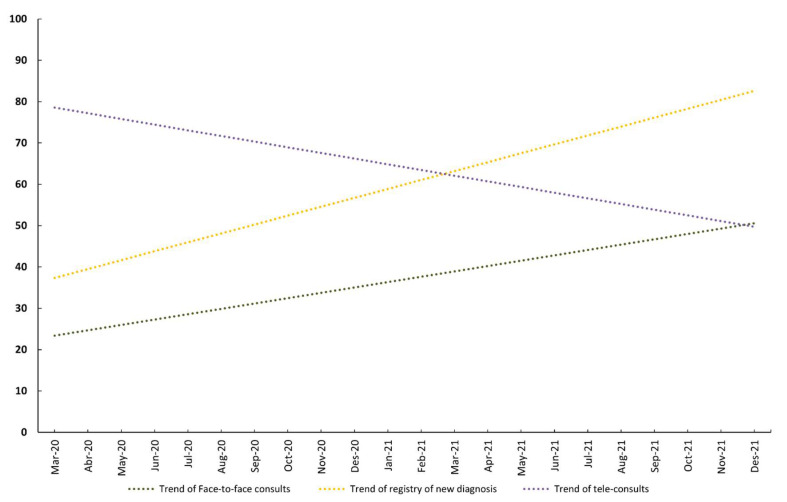
Trends in monthly new diagnoses (excluding COVID-19 cases), tele-consults and face-to-face consults, from January 2020 to December 2021.

The linear regression model of monthly face-to-face consults showed a positive, significant linear trend with a slope of 1.29 (95% CI, 0.49 to 2.1: *p*-value for trend = 0.004; R^2^ = 0.33). The linear trend of new records of diagnoses was almost parallel to the face-to-face consults trend, with a slope of 2.15 (95% CI, 1.39 to 2.92: *p*-value for trend < 0.001; R^2^ = 0.60). However, the trend of tele-consults was almost perpendicular to that of face-to-face consults and new records of diagnostic trends, with a slope of −1.37 (95% CI: −2.45 to −0.29: *p*-value for trend = 0.02; R^2^ = 0.23). Figure 3 shows the linear trends for face-to-face and tele-consults and new records of diagnoses during the pandemic period.

## 4. Discussion

We examined the evolution of types of PHC consults based on COVID-19 cases, PHC professionals, and diagnostic registry activity. The results showed that face-to-face consults were positively associated with GPs’ diagnostic registry activity, and tele-consults had no impact on the registry of new diagnoses, which is a crucial PHC function. The increase in tele-consults and the reduction in face-to-face consults were not proportional across age groups, with patients aged ≥65 years making less use of tele-consults. 

In 2020–2021, PHC consults increased by 24% compared with 2017–2019. In contrast, there was a rise of 45% in tele-consults by GPs. PHC studies also showed that tele-consults with GPs rose during 2021; New York City (USA) reported an 84.4% rise (including video calls, telephone, and e-consults) in the first COVID-19 wave, which fell by 32.7% in the fifth wave [23], while in New Brunswick (Canada), tele-consults increased by 113% during 2020 [24], and in Holland, telephone and e-mail consults rose from 30.6% in 2019 to 53.3% in March 2020 [25]. However, few studies reported rates of PHC tele-consults according to the type of professional. We found significant differences between professionals (e.g., tele-consults by PHC adult nurses only increased by 14.4%). These results may indicate that PHC nurses may not be able to change to tele-consults as easily as GPs. In Spain, nurses were responsible for the main COVID-19 diagnostic techniques (e.g., PCR testing) and in PHC, they restricted follow-ups to patients that required face-to-face consults (e.g., wound healing), which, in the study area, are prevalent given the aging of the population. However, when the public health authorities imposed more restrictions on PHC access due to high COVID-19 transmission, GPs were able to carry out part of their clinical activities with tele-consults, principally unscheduled spontaneous consults, and the continuity of care for patients with chronic diseases. In both cases, GPs and nurses restricted PHC activities to prioritize COVID-19 care. We suggest that, after the pandemic, tele-consults should be extended if they are more versatile or may remain as only a part of clinical activities, while more tele-consults might require further development to include PHC nurse activities that do not require face-to-face meetings with patients [26].

Consults in younger age groups rose 74% and 52% compared with 13% and 12% in older age groups. However, older people were more affected by COVID-19 [27] and also had more chronic conditions. This raises concerns about PHC restrictions during the pandemic that led to the replacement of face-to-face consults by tele-consults. A recent study found that mass mitigation strategies, such as closures of schools and day-care centers or limiting access to PHC, were of low utility given the enormous losses [28]. Temporary disruptions or changes in access to PHC services due to natural disasters have worsened the identification and follow-up of chronic conditions (e.g., hypertension) in vulnerable groups, such as older people [29]. Changing access to healthcare services has long-term health consequences in patients with chronic conditions [30]. A study before the pandemic reported 38% of patients aged >65 years old had problems in managing tele-consults or telephone calls [31]. Aging is associated with an increase in the digital divide. Older people require more PHC services, but are less likely to use telemedicine [32,33]. While some initiatives have made efforts to reverse the digital divide by offering technological devices and instruction to older people [34], the problem remains unresolved and is a source of inequity. Although advocates of tele-consults claim better disease control in hospital and specialized healthcare, PHC access is currently partially thwarted by patients’ ages [35] and in the years before COVID-19, others warned that implementing tele-consults might impede access for older people [36]. While COVID-19 seemed to increase therapeutic relationships between patients and PHC professionals through tele-consults, paradoxically, those who most need care preferred human relationships not mediated by technological devices.

The registry of new diagnoses was clearly associated with face-to-face consults. These results can be understood through daily clinical practice; diagnostic activity might be closely related to a face-to-face consult because physicians can verify symptoms, signs and other clinical findings in person. Follow-up and monitoring (including new treatments) begin when physicians first carry out and register new diagnoses. For example, a GP cannot prescribe a drug treatment to treat hypertension, nor refer patients to follow-up visits with PHC nurses without a diagnostic record of hypertension. In other words, PHC GPs’ diagnostic clinical activity and registry activates all care processes. A recent study in the same PHC centers during the first year of the COVID-19 pandemic showed that diagnostic records decreased [12], and the Catalonian Primary Care Services Information Systems also warned of a rapid and substantial decline in diagnoses of cardiovascular risk factors and other chronic diseases in the first three months of the COVID-19 pandemic—from February to April 2020 [37]. Our study also confirms that a decrease in face-to-face consults is associated with, and probably causes, a decrease in diagnostic activity [38].

Prioritization of COVID-19 in PHC and replacing face-to-face consults with tele-consults during the last two years (2020 and 2021) will have collateral health consequences. As PHC acts as the citizens’ gatekeeper to the healthcare system, late diagnoses of the most prevalent diseases [12] or worse control of chronic disease follow-up are only some examples [16]. Our results add to the evidence of the undesirable effects of making tele-consults the first option, specifically in PHC. Tele-consults are not appropriate for all patients, cannot replace face-to-face consults [39], and may delay referrals to other healthcare settings or specialists when needed [40]. We accept that the PHC digital era requires innovation to support individuals and should be technology-enabled, but one must not forget that PHC is community-based and patient-centered and that universal access enables social equity from the point of view of the health care system and is vigilant of the health of individuals when setting appropriate healthcare measures [38]. Our results suggest tele-consults, as currently implemented, may threaten access for the elderly.

The study has some limitations; firstly, the data came from three PHC centers out of a total of fifty-four in Barcelona. However, the PHC centers offer healthcare service to all citizens. In the year before the pandemic, 78% of all citizens make use of PHC services at least once, therefore ensuring the representativity of the population studied. Furthermore, all PHC centers in Catalonia followed a similar regime of restrictions regarding tele-consults and face-to-face consults because of the health situation derived from COVID-19 and directives from the Health Ministry; therefore, any differences are expected to be minimal. The study focused on associations between face-to-face consults and diagnoses, but not on the follow-up or monitoring activity, which would require other indicators. Given our limited data, the linear regression models used to estimate time trends of face-to-face, tele-consults and new diagnoses were only intended to compare slope estimates, but not to model events. However, our results suggest that further study using time series or interrupted time series would allow a more in-depth parameterization of the events of the COVID-19 years. The source of the data, which came from an urban area characterized by an aging population, might limit generalization to urban areas (not rural or semi-rural). For this study, the data were aggregated, and the number of variables obtained was limited, lacking information on sex, other sociodemographic characteristics, clinical factors, and patient satisfaction and preferences. These variables could be confounders or interacting factors between diagnostic registry activity and the type of consult. Our results highlight the need to study these factors in future studies to improve planning strategies and policies adapted to different population groups.

## 5. Conclusions

PHC tele-consult policies have not solved the heightened demand for PHC services brought on by COVID-19 disease. The pandemic resulted in the displacement of clinical activity, and tele-consults could not replace face-to-face activity unrelated to COVID-19. Tele-consult technology may require better tuning and alignment with PHC clinical tasks to include the most vulnerable population, specifically older people. Future policies and actions during disasters or public health emergencies require analysis and understanding of physicians’ and nurses’ clinical practices to modify tele-consults and contextualize the technology within the intrinsically human practice that medicine is and will continue to be.

## Figures and Tables

**Table 1:** Mean visits to PHC professionals during the pre-pandemic period and the two years of COVID-19. Visit ratios for number of mean visits in 2020 and 2021 compared with mean visits from 2017 to 2019.

Type of Visit	2017–2019	2020–2021	Ratio 20–21 vs. 17–19 ^1^	95% CI
Total	511,849	636,701	1.24	1.24 to 1.25
Face-to-face	411,126	293,541	0.71	0.71 to 0.72
Tele-consults	100,723	343,161	3.41	3.38 to 3.43
General practitioner	314,396	342,487	1.09	1.08 to 1.09
Face-to-face	241,440	107,046	0.44	0.44 to 0.45
Tele-consults	72,957	235,441	3.23	3.2 to 3.25
Primary care adult nurse	142,557	191,704	1.34	1.34 to 1.35
Face-to-face	122,236	136,771	1.12	1.11 to 1.13
Tele-consults	20,321	54,933	2.70	2.66 to 2.75
Pediatrician	24,710	52,896	2.14	2.11 to 2.17
Face-to-face	21,421	17,444	0.81	0.8 to 0.83
Tele-consults	3289	35,452	10.78	10.4 to 11.18
Pediatric nurse	14,203	33,707	2.37	2.33 to 2.42
Face-to-face	13,153	26,333	2.00	1.96 to 2.04
Tele-consults	1049	7374	7.03	6.59 to 7.51
Social worker	8390	7740	0.92	0.89 to 0.95
Face-to-face	5801	1881	0.32	0.31 to 0.34
Tele-consults	2590	5859	2.26	2.16 to 2.37
Dentist	7593	8169	1.08	1.04 to 1.11
Face-to-face	7076	4067	0.57	0.55 to 0.6
Tele-consults	517	4102	7.93	7.26 to 8.72

^1^ Ratios between means of consults of consults in 2017–2019 and 2020–2021.

**Table 2:** Visit ratios between 2020 and 2021 and mean visits from 2017 to 2019 according to age groups (15–44, 45–64, 65–74, and >74 years). Values are represented as visit ratios for the number of visits in 2020–2021, compared with mean visits in 2017–2019.

Type of Visit	15–44 Years	45–64 Years	65–74 Years	>74 Years
Total	1.74	1.52	1.13	1.12
Face-to-face	0.82	0.66	0.63	0.70
Tele-consults	9.45	6.32	2.99	2.30
General practitioners	1.50	1.40	1.02	1.01
Face-to-face	0.56	0.45	0.38	0.44
Tele-consults	8.60	5.88	2.88	2.26
Primary care nurse	2.92	1.93	1.37	1.28
Face-to-face	1.93	1.25	1.06	1.04
Tele-consults	16.64	9.01	3.45	2.32
Social Worker	1.76	1.60	1.09	1.13
Face-to-face	0.40	0.33	0.29	0.30
Tele-consults	6.77	5.85	3.01	2.84
Dentist	0.49	0.65	0.53	0.61
Face-to-face	0.33	0.49	0.44	0.51
Tele-consults	2.56	4.81	1.61	2.60

## Data Availability

The data that support the findings of this study are partially included in the Supplementary Material; the full dataset is available from the corresponding author upon reasonable request.

## Supplementary Materials

The following supporting information can be downloaded at: https://www.mdpi.com/article/10.3390/ijerph192114119/s1, Table S1: A table list of diseases analyzed and their ICD-10 codes; Table S2: Original data and rescaled values displayed in Figure 2.
