# Supplementary material for: Face-to-Face and Tele-Consults: A Study of the Effects on Diagnostic Activity and Patient Demand in Primary Healthcare
Source: Int J Environ Res Public Health. 2022 Oct 29;19(21):14119. doi: 10.3390/ijerph192114119 (PMC9656153; doi:10.3390/ijerph192114119)
Supplement: Supplementary file 1 [file ijerph-19-14119-s001.zip › ijerph-1936594-supplementary.pdf]

Supplementary material

Table S1. Supplementary material 1: a table list of diseases analysed and their ICD-10 codes

Disease	ICD-10 codes
Heart failure	I50.1, I50.2, I50.3, I50.4, I50.8, I50.9, I50.20, I50.30
Hypertension	I10
Type 2 diabetes	E11,
Hypercholesterolemia	E78.0, E78.1, E78.2, E78.3, E78.4, E78.5, E78.6, E78.7, E78.8, E78.9
Chronic kidney disease	N18, N18.1, N18
Stroke	I63.0, I63.1, I63.2, I63.3, I63.4, I63.5, I63.6, I63.7, I63.8, I63.9, I63.312, I63.89
Ischemic cardiomyopathy	I25.9, I24.9, I25.5, I25.6
Acute myocardial infarction	I21.9, I21
COPD	J44.0, J44.1, J44.9,
Asthma	J45.2, J45.3, J45.4, J45.5, J45.9, J45.902, J45.22, J45.21, J45.20
Osteoporosis	M81.0, M81.6, M81.8, M80
Dementia	R41.81, F02.0, F01, F03, G31.84
Hypothyroidism/thyroiditis	E06.1, E06.9, E06.6, E03.9, E03.1, E03.0,
Benign prostatic hypertrophy	N41.9, N41.1, N40
Anxiety	F41.9, F41.0, F41.1, F41.3, F41.8
Depression	F33, F32
Alcohol use disorder	F10.1, F10.2, F10.9
Violence against women	T74.11, T74.9, T74.2
Breast cancer	D05.0, D05.1, D05.8, D05.9, C50.919
Colon cancer	C18.9
Benign tumours of the colon	D12.0, D12.1, D12.2, D12.3, D12.4, D12.5, D12.6, D12.7, D12.8, D12.9
Prostate cancer	C61
Lung cancer	C34.0, C34.1, C34.8, C34.9
Bladder cancer	C67.0, C67.1, C67.2, C67.3, C67.4, C67.5, C67.6, C67.7, C67.8, C67.9
Liver cancer	C22.0, C22.1, C22.2, C22.3, C22.4, C22.7, C22.8, C22.9
Thyroid cancer	C75.0, C73
Melanoma	C43.0, C43.1, C43.2, C43.3, C43.4, C43.5, C43.6, C43.7, C43.8, C43.9
Stomach cancer	C16.0, C16.1, C16.2, C16.3, C16.4, C16.5, C16.6, C16.8, C16.9
Kidney cancer	C64.1, C64.2, C64.9
Lymphoma/Leukaemia	C91.A, C91.Z, C91.0, C91.1, C91.3, C91.4, C91.5, C91.6, C91.9, C92.A, C92.Z, C92.0, C92.1, C92.3, C92.4, C92.5, C92.6, C92.9

Table S2. Original data and rescaled values displayed in figure 2.

New diagnostic records	2020											
	**Jan-20**	**Feb-20**	**Mar-20**	**Abr-20**	**May-20**	**Jun-20**	**Jul-20**	**Aug-20**	**Sep-20**	**Oct-20**	**Nov-20**	**Dec-20**
Heart failure	16	15	14	8	7	24	6	12	7	12	10	28
Hypertension	54	57	34	20	31	25	25	22	26	32	35	27
Type 2 Diabetes	21	28	21	8	22	20	18	8	15	19	26	28
Hypercholesterolemia	67	61	38	17	21	32	44	19	38	41	57	63
Chronic kidney disease	14	13	14	5	10	24	25	5	14	24	30	32
Stroke	9	10	5	7	14	10	14	7	4	7	10	17
Ischemic cardiomyopathy	9	12	10	1	5	5	8	5	7	4	6	8
Acute myocardial infarction	4	12	6	5	4	4	2	3	13	8	6	5
COPD	8	12	9	1	2	4	7	1	4	17	12	11
Asthma	27	31	31	23	22	23	24	15	14	40	28	25
Osteoporosis	25	24	15	1	6	18	10	12	12	21	29	21
Dementia	41	43	22	19	22	36	33	26	33	41	40	52
Hypothyroidism/thyroiditis	34	41	16	3	9	17	22	16	21	16	21	19
Benign Prostatic Hypertrophy	44	34	15	4	12	22	23	14	20	35	27	74
Anxiety	127	154	157	168	100	137	123	98	132	119	136	113
Depression	42	42	25	15	23	18	25	25	31	28	35	37
Alcohol use disorder	10	9	3	2	0	4	3	4	5	6	3	3
Violence against women	5	5	2	3	2	3	2	3	1	2	2	3
Breast cancer	5	3	4	1	3	6	14	5	7	11	14	12
Colon cancer	5	5	2	1	7	2	3	1	6	4	5	2
Benign tumours of the colon	28	29	18	5	9	20	12	3	15	21	29	24
Prostate cancer	11	10	5	1	6	3	4	5	3	8	2	1
Lung cancer	1	2	4	1	4	5	4	6	6	3	4	7
Bladder cancer	6	7	3	1	1	4	3	2	0	3	5	7
Liver cancer	0	0	2	0	1	1	0	0	1	0	0	2
Thyroid cancer	0	3	1	0	1	0	0	0	0	0	1	0
Melanoma	4	3	2	0	0	3	3	1	3	4	1	1
Stomach cancer	0	0	0	0	0	1	0	1	1	2	0	2
Kidney cancer	1	1	1	2	1	3	0	0	3	2	1	2
Lymphoma/Leukaemia	0	2	0	0	0	2	3	0	1	1	0	2
**Sum (rescaled to 0-100)**	**618 (62.6)**	**668 (67.6)**	**479 (48.5)**	**322 (32.6)**	**345 (34.9)**	**476 (48.2)**	**460 (46.6)**	**319 (32.3)**	**443 (44.8)**	**531 (53.7)**	**575 (58.2)**	**628 (63.6)**

New diagnostic records	2021											
	**Jan-21**	**Feb-21**	**Mar-21**	**Apr-21**	**May-21**	**Jun-21**	**Jul-21**	**Aug-21**	**Sep-21**	**Oct-21**	**Nov-21**	**Dec-21**
Heart failure	18	30	25	20	18	24	14	11	21	22	30	22
Hypertension	42	45	60	54	45	49	40	22	36	68	86	59
Type 2 Diabetes	21	44	45	35	42	72	45	25	48	61	62	38
Hypercholesterolemia	71	72	98	75	105	83	72	54	70	91	100	68
Chronic kidney disease	19	29	37	34	39	40	42	27	23	45	51	24
Stroke	8	5	9	10	14	14	12	16	23	14	24	20
Ischemic cardiomyopathy	9	6	14	16	14	17	14	7	13	18	14	12
Acute myocardial infarction	10	8	7	7	12	13	14	11	22	14	22	10
COPD	6	11	7	15	12	13	11	5	7	13	27	14
Asthma	18	13	41	25	32	54	46	20	40	61	66	53
Osteoporosis	19	29	28	29	35	25	22	12	25	34	30	23
Dementia	25	26	21	18	20	33	22	12	33	5	5	4
Hypothyroidism/thyroiditis	18	30	32	26	23	29	24	12	25	35	42	30
Benign Prostatic Hypertrophy	27	34	43	38	57	31	39	23	42	59	70	45
Anxiety	132	137	135	147	158	152	131	96	179	168	169	111
Depression	39	41	50	47	50	63	37	23	57	60	77	42
Alcohol use disorder	11	9	7	4	11	11	8	11	13	11	16	7
Violence against women	1	2	4	4	5	4	4	2	4	6	5	3
Breast cancer	6	13	16	13	12	13	7	4	9	13	7	7
Colon cancer	4	7	6	3	1	1	7	2	7	6	7	4
Benign tumours of the colon	19	23	32	27	28	25	22	11	16	29	44	16
Prostate cancer	3	9	5	9	7	7	5	2	7	9	6	2
Lung cancer	3	4	2	6	5	9	4	8	6	4	5	2
Bladder cancer	3	3	5	4	5	3	3	3	5	7	9	7
Liver cancer	0	0	2	0	2	1	1	2	2	3	3	0
Thyroid cancer	2	1	2	2	1	0	2	2	1	2	2	1
Melanoma	2	1	4	4	5	3	3	2	6	3	2	2
Stomach cancer	0	1	0	0	1	1	1	3	1	1	1	2
Kidney cancer	2	2	1	2	2	4	1	1	0	0	5	1
Lymphoma/Leukaemia	0	0	0	2	2	0	0	0	2	1	1	5
**Sum (rescaled to 0-100)**	**538 (54.5)**	**635 (64.3)**	**738 (74.7)**	**676 (68.4)**	**763 (77.2)**	**794 (80.4)**	**653 (66.1)**	**429 (43.4)**	**743 (75.2)**	**863 (87.3)**	**988 (100)**	**634 (64.2)**

	2020											
	**Jan-20**	**Feb-20**	**Mar-20**	**Abr-20**	**May-20**	**Jun-20**	**Jul-20**	**Aug-20**	**Sep-20**	**Oct-20**	**Nov-20**	**Dec-20**
**COVID-19 cases (Rescaled to 0-100)**	NA ()	NA ()	387 (9.1)	257 (6)	174 (4.1)	108 (2.5)	343 (8)	450 (10.6)	393 (9.2)	1339 (31.4)	658 (15.4)	769 (18)
**Face-to-face consults (Rescaled to 0-100)**	21130 (100)	21060 (99.7)	12682 (60)	3209 (15.2)	3693 (17.5)	5209 (24.7)	6367 (30.1)	5650 (26.7)	6844 (32.4)	7503 (35.5)	6692 (31.7)	6783 (32.1)
**Tele-consults (Rescaled to 0-100)**	7135 (22.1)	7712 (23.9)	17280 (53.5)	23420 (72.5)	26883 (83.2)	28915 (89.5)	27242 (84.3)	15759 (48.8)	22152 (68.5)	32319 (100)	30127 (93.2)	25729 (79.6)

	2021											
	**Jan-21**	**Feb-21**	**Mar-21**	**Apr-21**	**May-21**	**Jun-21**	**Jul-21**	**Aug-21**	**Sep-21**	**Oct-21**	**Nov-21**	**Dec-21**
**COVID-19 cases (Rescaled to 0-100)**	1390 (32.6)	579 (13.6)	518 (12.2)	495 (11.6)	252 (5.9)	826 (19.4)	2965 (69.6)	439 (10.3)	180 (4.2)	74 (1.7)	420 (9.9)	4262 (100)
**Face-to-face consults (Rescaled to 0-100)**	6013 (28.5)	6196 (29.3)	7527 (35.6)	6866 (32.5)	7740 (36.6)	9092 (43)	9366 (44.3)	5387 (25.5)	8330 (39.4)	12698 (60.1)	15150 (71.7)	12905 (61.1)
**Tele-consults (Rescaled to 0-100)**	15919 (49.3)	18068 (55.9)	19942 (61.7)	17880 (55.3)	17789 (55)	16767 (51.9)	16369 (50.6)	9032 (27.9)	12328 (38.1)	19502 (60.3)	20770 (64.3)	21842 (67.6)
